# Cognitive function in soccer athletes determined by sleep disruption and self-reported health, yet not by decision-reinvestment

**DOI:** 10.3389/fneur.2022.872761

**Published:** 2023-02-06

**Authors:** Jasmin Pourhassan, Jane Sarginson, Wolfgang Hitzl, Kneginja Richter

**Affiliations:** ^1^Department of Life Sciences, Faculty of Science and Engineering, Manchester Metropolitan University, Manchester, United Kingdom; ^2^University Clinic for Psychiatry and Psychotherapy, Klinikum Nuernberg, Paracelsus Medical University, Nuremberg, Germany; ^3^Department Research and Innovation Management (RIM), Biostatistics and Publication of Clinical Trial Studies, Paracelsus Medical University, Salzburg, Austria; ^4^Department of Ophthalmology and Optometry, Paracelsus Medical University, Salzburg, Austria; ^5^Research Program Experimental Ophthalmology and Glaucoma Research, Paracelsus Medical University, Salzburg, Austria; ^6^Faculty for Social Work, Technical University for Applied Sciences, Nuremberg, Germany; ^7^Faculty for Medical Sciences, Goce Delcev University, Stip, North Macedonia

**Keywords:** sleep disruption, soccer athletes, pitch performance, physical health, mental health, reinvestment, decision making, reaction-time (RT)

## Abstract

**Background:**

Sleep disruption (SD) increases sympathetic activity and cortisol secretion, and delays cognitive functions such as reaction-time (RT). Sympathetic activity of disturbed sleepers, is similar to those of so-called decision-reinvesters. Decision-reinvestment refers to traits in individuals with greater tendency to ruminate and reinvest in their decisions, with significant decrease in both motor-control and cognitive performance. Decision-making quality is a crucial attribute to athletic performance which relies on RT. Consequently, SD affects pitch-performance negatively, particularly in decision-reinvesters. This observational pilot-study examined the relationship between SD and cognitive function, perceived health, as well as reinvestment strategies. The hypothesis was that athletes with lower SD perceive their health better, report lower stress levels, perform better in cognitive tasks, and show lower tendency for decision-reinvestment.

**Methods:**

Twenty-one football player recorded their sleep with fit-trackers for 7 nights. Participants self-reported their mental and physical health, decision-reinvestment strategy, sleep behaviour, and perceived stress levels. Athletes then performed a set of cognitive tests to examine memory function (Backwards Corsi), selective attention (STROOP), and cognitive flexibility (Wisconsin Card Sorting Test, WCST). Normality was tested with a Shapiro-Wilk test, and analysed with a Pearson's or Spearman's correlation test.

**Results:**

Significant correlation appeared between extended sleep-interruptions and Backwards Corsi RT, r = 0.66, *p* = 0.010, as further in total sleep time and wellbeing r = 0.50, *p* = 0.029. A negative correlation exist in regard of pain scores and Backwards Corsi scores r = −0.57, *p* = 0.110. Physical health correlated with error-rates in the WCST, r = 0.69, *p* ≤ 0.001. Also, reinvestment negatively correlated with physical health, r = −0.80, *p* ≤ 0.001.

**Conclusion:**

Wellbeing relies on total sleep-time. Athletes with extended sleep-interruptions are slower in recalling memory, and those with greater reported pain have lower memory scores. Participants who rate physical health greater, have more error-rates in the WCST; indicating that cognitive flexibility is enhanced in individuals with inferior perceived health. However, individuals with lower physical health scores also have greater tendency to ruminate and reinvest in decisions, suggesting interrelation between reinvestment and physical health.

## Introduction

Cognitive performance refers to the ability to execute either one single- or a set of adequate action in response to external stimuli, in short amount of time. Tasks in this regard may include logical reasoning, mental addition, and other simple cognitive tasks (1). Yet, decision making requires great amount of tactics, metacognitive skills, mental simulation and team skills; and is therefore considered a complex cognitive task (2), and is a key component for athletic performance on the pitch (3). Competitive athletes are constantly required to decide about stimuli response with regard to their limited physiological energy resources to outperform fellow athletes, also known as pacing (4). Especially in high-impact sports such as football, foregoing any chance to physically power-out, can potentially be a missed score, or even worse an advantage for the opponents; and therefore requires athletes to make quick decisions under enormous pressure during competitions (5).

Performance in decision making tasks however is negatively affected in individuals with poor sleep (6). Telzer and his colleagues (6) came to the conclusion that poor sleep not only worsen cognitive performance, but also increases risk-taking behaviour in adolescence. Participants with lower reported sleep in the Pittsburgh Sleep Quality Index had less dorso lateral prefrontal cortex (DLPFC) activation, and increased activity of the anterior insula cortex (AIC) during reward processing in decision making tasks (6). The function of the AIC is to process bodily sensations in regard of emotions, and is used to evaluate normative social behaviours, as well as risks (7), and therefore influences decision making. Telzer further claims that bad sleepers also perceived greater positive consequences when taking risks. Findings in regard of cognitive performance decline can be also confirmed by Killgore et al. (8) who came to similar conclusion, investigating the effect of 49.5 h of sleep deprivation in 34 healthy adults. Participant's decision making performance in the Iowa Gambling Task (IGT) in a repeated measure study, was significantly affected by sleep disruption (SD) in the Killgore study. The authors further suggests that decision making under conditions of uncertainty, such as in the IGT, may be even more pronounced with increased age in sleep deprived adults.

**Figure 1:**
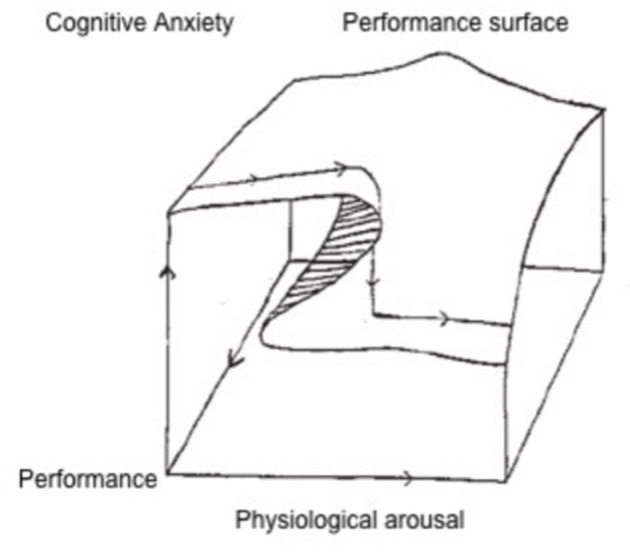
Chaos theory (10). The performance surface relies on the integration of both cognitive anxiety and physiological arousal. While on the back the performance surface is barely affected by physiological arousal during low of cognitive anxiety; the front of the performance surface significantly drops during high cognitive anxiety when the physical arousal is great.

Competitive concerns however are known stressors (5) and a source of threat per definition “… as the athlete's image is usually associated with his or her performance, the final result is always uncertain, there is exposure to public opinion and judgement by third parties, among others” (9). According to the chaos theory of Hardy and Fazey (10), cognitive stress only affects performance negatively, if the physiological response is great. Therefore, high cognitive stress does not necessarily influence pitch-performance negatively, if the physiological arousal is low; only once the physiological response to stress raises, performance regression may occur (Figure 1).

Similar findings were also observed in a study of Mosley et al. (11), that compared HRV measures of 49 participants performing the Automated Version of the Operation Span Task (AOSPAN), both at rest and under pressure, and examined working memory. The team around Mosley previously claimed that participants with greater cardiac vagal activity had performed better (12), even though the researchers could not confirm this theory in their latter research. HRV is a reliable measure to access cardiac vagal activity and regeneration status in athletes (13), and commonly used to adjust training workload accordingly and maximise training efficacy. With regard to changes in brain activity of individuals with poor sleep (6), suspicions arise that individuals with greater vagal activity at rest perform better under pressure (11) because they regenerate better (13); as indicated by correlations with changes in HRV (14). Interestingly, the experimental study of Dulleck et al. (14), examined HRV measure in comparison with brain scans during decision making tasks, and found sympathetic response to correlate with brain activity in the DLPFC and AIC during reward processing, regardless of sleep. These mentioned brain areas are identical with those that Telzer claims to be significantly altered in individuals with poor sleep (6). Therefore, differences in HRV may also be an indicator for diffrences in sleep.

Competitive concerns whatsoever, potentially lead to greater sympathetic response in athletes with disrupted sleep, and further increase anxiety related physical arousal; and consequently affect cognitive performance negatively (10, 12). In this regard, Usui and Nishida (15) examined the lasting effect of stress induced changes in HRV. Participants in the Usui study performed the STROOP colour-word test in the local language, and HRV measures were taken at rest, during the task, as well as every 15 min after the task for 2 h; and further analysed with a spectrogram. The STROOP colour-word test is a validated stressor that increases cortisol levels and sympathetic response of the autonomous nervous system (16). The sympathetic nervous system is responsible for vasoconstriction, and therefore in charge to raise blood pressure and heart rate, as well as to contract the external intercostal muscles and diaphragm; but also triggers the hypothalamic-pituitary-adrenal (HPA) axis to support and maintain increased blood pressure by secreting cortisol (17). Consequently, sympathetic activity correlates with changes in HRV (14). The STROOP test requires participants to process a specific stimulus feature while processing an interfering stimulus, such as identifying the colour of the word “green” in blue fonts. Colour detection is a non-automated skill and requires selective attention, and consequently causes stress, and therefore an increase in processing time and drop in accuracy; also known as the STROOP effect (18). A stress induced decrease in the very-low frequency (VLF) band (0.04–0.0033 Hz) of HRV could be observed in participants in the Usui research during the STROOP test, and regression in the VLF of HRV remained significantly low even 2 h after applying the stressor. A low frequency power of the VLF band is generally associated with increased chronic inflammation, metabolic syndromes, and prognosis of cardio vascular disease, whereas a high power is linked to high exercise capacity (19). This would indicate that repetitive competitive concerns could potentially steadily trigger sympathetic activity, and maybe exaggerate sympathetic stress response in athletes with poor sleep patterns, and consequently affect cognitive performance on the pitch negatively.

For the purpose of identifying and addressing sleep related areas that could potentially be a threat to athletes' physical- and mental- regeneration, Driller et al. (20) develop the Athletes Sleep Behaviour Questionnaire (ASBQ). The ASBQ differentiates between sport and competition related concerns, routine/environmental related components, and behavioural related factors that may affect athletic performance. Each area can then be separately addressed to improve sleep, and in further sequence performance outcomes on the pitch. Mosley and her colleagues however claim that these trait and behavioural related differences in individuals seem to determine physiological response, as individuals with greater mental resilience, as well as have greater vagal activity, both at rest and under pressure (11).

Trait related performance differences under pressure were initially observed by Masters (21), who later on invented a six-point Likert scale, to identify individuals with greater tendency to experiment and reinvest in the execution of learned and automated motors skills under pressure (22). Kinrade et al. (23) modified Masters' Movement Specific Reinvestment Scale more decision specific, and came to the conclusion that the Decision Specific Reinvestment Scale (DSRS) was an even greater predictor to identify athletes who will fail to perform automated motor skills, like throwing a basketball free-shots, when under high pressure; and refers to those individuals as high-reinvesters. The DSRS however is a five-point Likert scale, and consists of 13 statements, that evaluates conscious processing in regard to decisions-making and previous poor decisions. Statements are reflected on a 0 (“extremely uncharacteristic”) to 4 (“extremely characteristic”) continuum. Individuals with higher scores, have an increased tendency to ruminate and reinvest in their decisions. Interestingly, Williamson et al. (24) suggested in the same year that the neuroanatomical structure used in the anticipatory response to exercise effort is basically the same as in emotion, stress, and pain. Williams suggestion could potentially explain why the DSRS was an even greater predictor for performance decline than the MSRS. Williamson further claims that both behavioural- and exercise neuroscientist “… may have been observing the same thing over decades from different sides of the same coin.” Laborde et al. (25) builds up on this finding and claims that low decision-reinvesters had more effective coping patterns in comparison to high decision-reinvesters, and suggests to examine the neurophysiological structure behind their observation. Mosleys' finding in 2018, as previously mentioned, later on provided evidence for differences in HRV of high-reinvesters, both at rest and under pressure, and claims that individuals with greater vagal activity at rest had less tendency to ruminate and reinvest (11). However, repetitive competitive concerns, as observed in high-reinvester athletes, and certainly increased sympathetic response as indicated by alternations in HRV could potentially not only link to reinvestment strategies (11); but also be linked to an impaired ability to rest well at night (6, 13, 14). Whether HRV differences in high-reinvesters link to amount of time spent in different sleep stages remains unknown at this point.

While trait anxiety is part of the personality that influences behaviour, state anxiety is an ambivalent mood component that adapts behaviour in response to situational demands (26). Competitive concerns however are known triggers for state anxiety, and characterised by arousal, or physical activation (somatic anxiety), alongside nervousness, worrying, and apprehension (cognitive anxiety). In sport settings state anxiety has been linked to both enhanced as well as decreased pitch performance (27). While some athlete benefit from certain amounts of physical arousal and nervousness, others fail to perform when getting over anxious (28). Worrying, and conscious processing however are known to decrease pitch performance effectiveness and efficiency (29), and may negatively influence individuals perception of control under pressure (30). Therefore, high-reinvesters are at greater risk to be more affected by state anxiety in regard of their performance, especially under great somatic activity (23). According to the Attentional Control Theory (ACT), human behaviour is governed by the top-down system, lead by knowledge, expectations and goals contents from the working memory; and the bottom-up system, in search for salient stimuli and potential threats in the environment (31). High-reinvesters tend to cope with potential threats by shifting attention control towards an internal attention focus and conscious information processing (21, 22), and therefore lower capacity in the working memory (32, 33). Eysenck and his colleagues claim in this regard, that according to the explicit-monitoring theory internal attention focus occupies the working memory, and therefore favours the bottom-up system; and further affects gaze behaviour in high-reinvesters towards the source of threat (34), rather than the solution. Opposing to the explicit monitoring theory, the distraction theory indicates that working memory capacities decrease when individuals attempt to split attentional focus between executing a motor task and worrying about the outcome of the performance, by creating a dual task-scenario (35). Whether it is internal attention focus, or a dual task scenario, both impair working memory capacities, and nurture the bottom-up system. Considering the theory that individual with SD have increased sympathetic response and increased cortisol secretion that is already retarding the working memory; the shifting attention towards the threat or conscious information processing would in any case further exponentiate the effect of an impaired working memory and the ability to make quick decisions under pressure, especially in high-reinvesters under high arousal.

Even though baseline working memory did not differ in high-reinvesters in comparison to low-reinvesters at rest (36), it does change under pressure and physical arousal (23). The Young paper examined working memory capacity in high-reinvesters at rest, using the Corsi block test. Participants are presented nine blocks in the Corsi test, and the examiner starts manually tapping on three. Individuals are then asked to imitate the sequence. If successful the examiner repeats this procedure multiple times, and increases the length of the sequence by one block. A computed version of the Corsi test was conducted in a study of Vandierendonck et al. (37) who compared the Corsi test performed both forwards and backwards, during a dual task condition. The authors came to the conclusion that performance regression was greater in the backwards Corsi test when performing a secondary task, than in comparison with the forwards Corsi. Also, cognitive performance and memory function decreased in a study of Lafortune et al. (38) conducting a similar test battery, the day after shorter durations of both REM and deep sleep. Correlations of sleep quality in regard of reinvestment strategies and cognitive function however has previously not been investigated. However, athletic performance capacity increases by the amount of supra-threshold stimuli, with regard to a proportionate scope of exercise-regeneration, including sleep (39). Overstimulation in this sense occurs when a recovery period interferes with a subsequent stimulus, and can negatively affect both physical and cognitive performance (40). Therefore, a critical part of pitch-performance is sufficient bed-time and rest.

Sleep is a “… highly organized [sic] state generated by the cooperative interplay of many behavioral [sic] and neural components” (41), and is the most powerful recovery tool known to science. The key impact of sleep is to enhance muscle recovery, through protein synthesis and human growth hormone (somatropin) release; but also the improvement of information processing and learning, by recalling and storing information for the long-term memory (42). The key purpose and function of sleep however remains not fully understood. From a historical point of view, periods of active sleep are marked by rapid eye movements (REM), that alternate with calmer non-REM sleep periods (43). The review of Peever and Fuller (42) updated the current understanding about REM sleep; by examining a range of recent work investigating the mechanics, functional and conceptual developments of sleep across different species, including humans. The paper reports that the use of modern neuroscientific tools allows more precise examination of brain areas that are linked with REM sleep control, and promote identifying underlying neurological processes in the body, that determine both physical and cognitive performance. REM sleep however, only makes up a small proportion of night sleep; and contributes in quantifying sleep quality; alongside awakelike brain activity, skeletal motor atonia (active suppression of skeletal muscle activity) with intermittent muscle twitches, oculomotor muscle activity, autonomic and respiratory activation, fluctuation of brain- and body temperature, as well as elevated arousal threshold (42). Sleep phases are progressively categorised in wakefulness, transition phase (form wakefulness into the next stage), light sleep, deep sleep, and REM sleep (44). Sleep quality is defined by the amount of time spent in each stage, and the ability to sleep straight through the night. Prolonged periods of awake time during night sleep are known as sleep disruption (SD). Both REM sleep and deep sleep had been awarded significant importance in regard of cognitive performance, and have shown to affect information processing (38). The team around Lafortune examined the relationship between sleep patterns and cognitive function, and suggested that disruptions in REM sleep might be an indicator of “… changes in acetylcholine transmission, which plays a role in new information encoding.”

Consequently, EEG, electrooculogram (EOG), and electromyogram (EMG) are popular techniques to quantify sleep, and determine the different stages of sleep in a clinical setting. The combined use of these techniques is also known as polysomnography (PSG). PSG is a multi-parametric and cost-intensive sleep-test that also includes the use of an electrocardiogram (ECG), nasal pressure transducer and thermistor, chest and abdominal movement belts, transcutaneous (CO2) monitor, and pulse oximeter (41). Consumer sleep trackers on the other hand only measure individuals' atonia and in some cases heart rate, and process the collected data as part of an algorithm to estimate sleep phases. The review of both Evenson et al. (45) and Svensson et al. (46) came to the conclusion that the reliability of consumer trackers for healthy sleepers is reasonably good in comparison to the golden standard PSG, but is lacking steadiness in identifying periods of insomnia (when individuals are awake but not moving), and falsely assume these to be periods of sleep in the majority of studies. Consumer trackers however are an affordable option for healthy sleepers to record sleep in their ordinary sleeping-environments over longer periods of time; and thereafter overcome the major limitations of PSG, which is mainly performed in a clinical setting. However, consumer trackers such as Apple Watch could be an acceptable alternative to the Philips Actiwatch for sleep monitoring (47).

Disruption of sleep may promote an exaggerated physiological response to stress (48), and individuals with reduced mental resilience as well as greater reinvestment tendency seem to be more affected by sympathetic activity during pressured tasks (11). Fang et al. (49) found no evidence for cognitive impairment in individuals suffering SD, but could find a tendency, though not significant, for a reduced high-frequency (0.15–0.40 Hz) band of HRV at rest, indicating decreased cardio vagal activity; as well as raised trait anxiety scores. Individuals in the Fang study were asked to perform four paper-pencil tasks in regard of sleepiness, anxiety, fatigue, and concentration difficulty before performing the Wisconsin Card Sorting Test (WCST). Resting HRV however was recorded under paced breathing. The WCST is a computerised test, and consist of 128 cards with geometric figures that vary in colour, form, and number. Participants are asked to find out how to match a given card according to these attributes, to one of four displayed cards on a screen, and receive immediate feedback. Successful strategies can be repeated, but classification rules change every 10 cards. Individuals are neither informed when the rules change, nor to what principle cards need to be sorted. The test ends when participants have placed 64 cards in one category or, otherwise, have sorted all 128 cards. The WCST examines prefrontal executive functions, and measures how well-individuals adapt to changing rules (50).

In conclusion, a disruption of sleep negatively affects athletic attributes such as reaction time, whereas evidence in regard of accuracy has been conflicted. Consumer trackers however allow to access relevant sleep stages in this regard, in individuals ordinary sleep-environments, over longer periods of time. High-reinvesters have previously shown significant alternations in sympathetic response under pressure, that correlate with changes in brain activity in areas that have also been identified in individuals with SD. The question whether physiological differences in high-reinvesters link to amount of time spent in different sleep stages remains unknown at this point.

The aim of this observational pilot-study therefore was to investigate how sleep patterns influence cognitive performance outcomes, and whether potential correlations can be linked to differences in trait. The hypothesis was that (I) low-reinvesters sleep better than high-reinvesters, (II) athletes with less SD have greater cognitive performance capacity, (III) perceived sleep quality as well as physical- and mental health is greater in athletes with less SD, and (IV) perceived stress score is higher in athletes with greater SD.

## Method

The study protocol was designed to fit the uncertainty of the time, and ongoing social distancing guidelines of the Fédération Internationale de Football Association (FIFA) in regard of external club members.

### Participants

Twenty-one male and female soccer athletes on club level of the first to fourth league of the Austrian Football Association (ÖFB) were recruited *via* the word of mouth for this study. Players had sufficient understanding of written and verbal English in order to provide full inform consent, perform questionnaires, and follow task relevant instructions. Inclusion criteria were (1) male and female soccer athletes, (2) between 18 and 40 years, (3) on club level (4) willing to use wearable technology or online physical activity tracking tools, to provide their collected data on their activity and sleep patterns. Exclusion criteria were (1) respiratory disorders, including asthma if not controlled; (2) cardiovascular issues, including uncontrolled hypertension, heart problems etc.; (3) diabetes type 1 or type 2, or any metabolic condition affecting the ability to perform sustained exercise, (4) recurring joint, bone or muscular injury or condition, (5) having had exercise testing in the previous 2 years that was terminated prematurely for reasons of health or safety, (6) any other disorder, complaint or injury disclosed to the investigators which may put the participant or an investigator at risk, or may affect the result of the study; (7) inability to participate in one or more of the tasks described in the study, or if it could be potentially detrimental to do so; (8) regular medications or used recreational drugs that might affect the results, or exacerbate a condition that is being treated; (9) cardiac pacemakers fitted, or any metallic implants (such as those used in bone surgeries etc; (10) currently undergoing or seeking treatment for a psychiatric disorder; (11) currently undergoing or seeking treatment for a medical condition that affect the ability to achieve restful sleep, such as insomnia and sleep apnea. Participants were screened and preselected *via* informal interviews to ensure eligibility for inclusion. Interview was led by a general health questionnaire.

### Apparatus

The wrist activity trackers used were: Withings Move (*N* = 11), Apple Watch series 2 (*N* = 1), series 5 (*N* = 2), series 6 (*N* = 1), either using Apple Health App (*N* = 3) or Pillow App (*N* = 1); Huawai band 6 (*N* = 1), Galaxy Watch active 2 (*N* = 1), Suunto 5 (*N* = 1), Garmin Venu (*N* = 1). Participants that did not feel comfortable wearing a wrist tracker over night, provided a sleep diary (*N* = 2).

### Procedure

Participants were electronically provided a participant information sheet (PIS) *via* MS Forms, that has previously been explained to them, and were instructed to ask questions at any time. After providing informed consent online, athletes were invited to (1) extract and send in a 7 days track of their fit tracking device. Tracking period were 7 consecutive days, starting on enrolment day. Athletes then received a link to the online platform PsyToolkit, which is a software package for programming psychological experiments using Linux (51, 52); to perform the (2) Health Questionnaire SF-36, to examine athletes' general mental and physical health status; (3) the Athletes Sleep Behaviour Questionnaire, to examine sleep behaviour (20); (4) Perceived Stress Level Questionnaire, to perceive stress levels of athletes, and determine potential modifier of cognitive performance (53); and (5) the Decision Specific Reinvestment Scale, to investigate decision making strategies under pressure (23). Questionnaires were followed by (6) the Backward Corsi Task, to test memory function (36); (7) the STROOP Test, to test selective attention performance, and to raise stress levels (15); as well as the (8) the Wisconsin Card Sorting Test, to check for cognitive flexibility (49), which because of the STOOP can be considered a pressured task. Athletes were in-season, and had no trans-meridian travel. Trainings status has not been recorded, but is typically between 3 and 5 days and 1 match day per week on average in these leagues. Evaluation took place in a 2 month total timeframe.

### Measures

The measures considered were respectively, (1) sleep quality, namely (a) total time spent after sleep onset time, including (b) light sleep, (c) deep sleep, (d) SD time, as well as (2) SF-36 total scores, each for (a) physical health, and (b) mental health; (3) ASBQ overall scores including: (a) sports-, (b) routine/environmental-, and (c) behavioural related scores; (4) Perceived Stress Level score; (5) DSRS overall scores, and each for the (a) decision-rumination, and (b) decision-reinvestment aspect; (6) Backwards Corsi (a) best score, and (b) mean RT, (7) STROOP (a) mean RT for successful attempts, (b) mean RT for failed attempts, (c) number of successful attempts; and finally the (8) Wisconsin Card Sorting (a) error-rates in percent, as well as (b) overall mean RT.

### Data analysis

The data was then analysed as the following: Seven days means of total sleep time, light sleep, deep sleep, and SD time were calculated using MS Excel. REM sleep was not provided in all data sets. In order to normalise deep sleep durations, in devices that had recorded REM sleep separately, and therefore had significantly lower durations of deep sleep; times spent in REM were assigned to deep sleep for the analysis (*N* = 4), as both sleep stages have similar qualities and the highest arousal threshold (54). Apple watches coupled with Health App (*N* = 3) only reported time spend in bed, and total sleep time. Data sets from Health App participants were therefore excluded when analysing for light sleep, deep sleep, and SD times, but included when analysing total sleep time. Same applies to data provided in sleep diaries, that were left out in these calculations for the same reason. Also, the entire data set recorded with the Huawai tracker (*N* = 1) was excluded when analysing SD times, as measures were not provided either (Table 1).

**Table 1:** Overview functional differences in used devices.

**Device**	**Total sleep**	**No. awake**	**Awake time**	**Light sleep**	**Deep sleep**	**REM sleep**	**In bed**
Withithings move	✓	✓	✓	✓	✓		✓
Apple watch (health app)	✓						✓
Apple watch (pillow app)	✓	✓	✓	✓	✓	✓	✓
Suunto 5	✓		✓	✓	✓		
Garmin venu	✓		✓	✓	✓	✓	
Samsung galaxy active 2	✓		✓	✓	✓	✓	
Huawai band 6	✓	✓		✓	✓	✓	
Sleep diary	✓						

Depending on the device and apps used, following of the requested sleep data was provided by participants.

Bivariate correlations were then calculated for light sleep, deep sleep, SD time, and total sleep time using SPSS, and correlated for each measure: SF-36 scores for (a) physical health, and (b) mental health; ASBQ overall scores; Perceived Stress Level score; DSRS overall scores; Backwards Corsi (a) best score, and (b) mean RT; STROOP (a) mean RT for successful attempts, (b) mean RT for failed attempts, (c) number of successful attempts; and the Wisconsin Card Sorting (a) error-rate in percent, and (b) mean overall RT; using the Pearson's test for parametric data, and Spearman's for non-parametric data. Normal distribution in parametric data was tested with a Shapiro-Wilk test. Further, SF-36 scores for (a) physical health, and (b) mental health; ASBQ overall scores; Perceived Stress Level score; DSRS overall scores were correlated to each measure of the Backwards Corsi (a) best score, and (b) mean RT; STROOP (a) mean RT for successful attempts, (b) mean RT for failed attempts, (c) number of successful attempts; and the Wisconsin Card Sorting (a) error-rate in percent, and (b) mean overall RT. Overall scores of the ASBQ were also correlated with Perceived Stress Level score. Participants with total DSRS scores of ≤ 26 were assigned to the low-reinvesters group, and participants with scores ≥ 27 were assigned to the high-reinvesters. Moreover, this method was applied to both the rumination (7 statements) and reinvestment (6 statements) aspect as well. Therefore, low-rumination was cut at ≤ 14, and low-reinvestment at ≤ 12. Significance level were set at r ≥ 0.5, *p* ≤ 0.05.

### Ethics

The project has been conducted under the academic ethical framework of the Manchester Metropolitan University. Favourable ethical opinion was granted under the reference number 25719 by the Science and Engineering Research Ethics and Governance Committee. Ethical concerns only existed for the STROOP test in regard of minor psychological manipulations, that were addressed with a pre-selection and exclusion of participants who could possibly be affected by underlying conditions, by performing a general health questionnaire upon recruiting. The University is registered with the Information Commissioner's Office (ICO), and manages personal data in accordance with the General Data Protection Regulation (GDPR) and the University's Data Protection Policy, and therefore does not share any personal data collected in this study with any third parties.

## Results

The data of all male (*N* = 13) and female (*N* = 8) participants was taken into account for the analysis. Participants were 25 years old (SD 7), yet age was not normally distributed. Participants slept 433.2 min (SD 49.5), consisting of 292.1 min (SD 43.5) light sleep, with an awake time of 26.4 min (SD 14.4) per night on average. The avarage scores on the SF-36 for physical and mental health were 1,756 (SD 373) and 1,140 (SD 153), respectively. Athletes scored 42 (SD 7) on avarege in regard of the sleep habits in the ASBQ, while Percived Stress was rated 22 (SD 6), and reinvestment 24 (SD 9). Individuals could recall 5 (SD 3) sequences with a RT of 250 s (SD 67) in the Backawrds Corsi test. STROOP performance resulted in 11 (SD 3) right attampts with an avarrage RT of 883 ms (SD 231). Error-rate in the WCST was 12 (SD 7) with a RT of 1692 ms (SD 591). With exception of one participant (*N* = 1), English was no-ones else's first language. Some participants (*N* = 2) did not successfully performed the test battery on PsyToolkit, and feed-backed high levels of stress, and not feeling ready to perform. Participants however did not withdraw from the study, and were therefore assigned the maximum stress score for the Perceived Stress Level test. Available data was considered for the analysis as previously explained in the Data Analysis section of this paper. Correlations are presented thematically in the following section, and further illustrated.

### Sleep and reaction time

**Figure 2:**
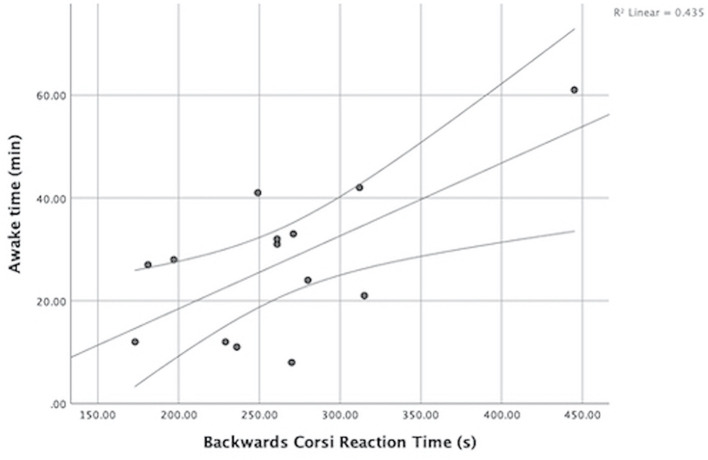
Relation between awake time during night sleep (min) and mean RT (s) during the Backwards Corsi test. Athletes with little amount of interrupted sleep were faster in recalling memory.

SD time and Backwards Corsi mean RT were both normally distributed (*p* > 0.05). There was a significant correlation between SD time (min) and Backwards Corsi mean RT (s), r = 0.66, *p* = 0.010 (Figure 2); suggesting that athletes with great amount of interrupted sleep were slower in recalling memory.

### Sleep and perceived health

The amount of total sleep (min) correlated with well being (mental health) scores of the SF-36 r = 0.50, *p* = 0.029. All other physical or mental health measures of the SF-36 were not influenced by any aspect of sleep r = ≤ 0.5, *p* ≥ 0.05.

### Perceived health and memory

A negative correlation exist in regard of SF-36 pain scores and CORSI scores r = −0.57, *p* = 0.110. Participants with greater pain scores had lower memory function.

### Perceived health and cognitive flexibility

**Figure 3:**
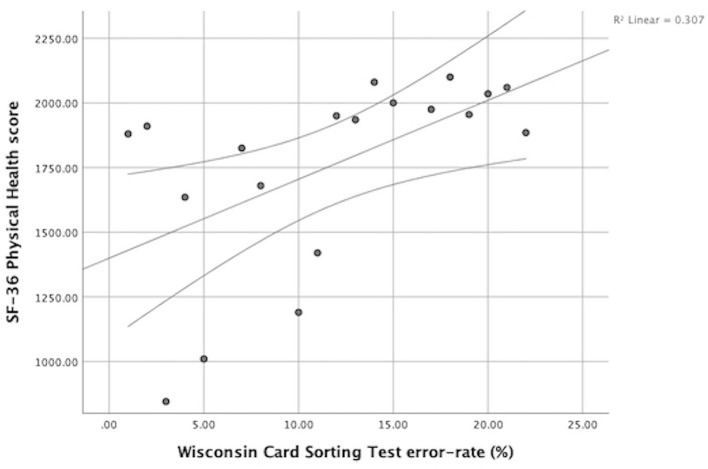
Relation between SF-36 Physical Health score and WCST error-rate (%). Athletes with decreased self-reported health (lower scores) had lower error-rates.

There was a clinically significant correlation between SF-36 physical health scores and Wisconsin Card Sorting error-rates r = 0.69, *p* = 0.001, (Figure 3), indicating that participants with greater self-reported health (higher scores) had an increased error-rate (%) in terms of cognitive flexibility.

### Perceived health and decision-reinvestment

**Figure 4:**
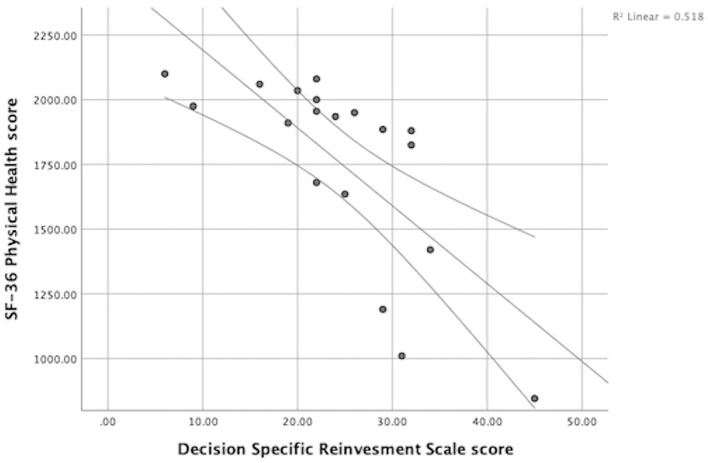
Relation between SF-36 Physical Health score and Decision Specific Reinvestment scores. Athletes with better self-reported health (greater scores) had less tendency for decision-reinvestment.

SF-36 physical health scores were also discovered clinically significant when correlated with DSRS scores, r = −0.80, *p* < 0.001 (Figure 4). Physical health, r = −0.75, *p* < 0.001, correlated slightly higher in the rumination aspect of decision-reinvestment; in comparison to the reinvestment aspect r = −0.65, *p* 0.003; presuming that individuals with greater self-reported physical health (higher scores) had less tendency to ruminate and reinvest in decisions.

Sleep whatsoever did not correlate (Table 2) with any other measure such as perceived stress, perceived sleep (ASBQ), and DSRS scores; and neither with cognitive flexibility (WCST), selective attention (STROOP), memory (Backwards CORSI) function.

**Table 2:** Correlation matrix of sleep vs. cognitive function and perception of stress, health and decision reinvestment.

**Cognitive function and perception of: Stress, health, reinvestment**	**SLEEP duration (min)**			
	**Total sleep**	**Awake time**	**Light sleep**	**Deep sleep**
Perceived stress	r = −0.01, *p* = 0.953	r = 0.17, *p* = 0.542	r = −0.09, *p* = 0.753	r = 0.42, *p* = 0.108
DSRS score	r = 0.04, *p* = 0.882	r = −0.04, *p* = 0.895	r = −0.13, *p* = 0.654	r = 0.27, *p* = 0.358
ASBQ score	r = −0.36, *p* = 0.581	r = −0.41, *p* = 0.146	r = −0.38, *p* = 0.177	r = 0.18, *p* = 0.550
Backward Corsi score	r = 0.29, *p* = 0.222	r = 0.04, *p* = 0.891	r = 0.09, *p* = 0.759	r = −0.15, *p* = 0.617
STROOP RT wrong (ms)	r = −0.28, *p* = 0.244	r = 0.00, *p* = 0.994	r = −0.15, *p* = 0.610	r = −0.14, *p* = 0.626
RT right (ms)	r = −0.33, *p* = 0.165	r = −0.19, *p* = 0.527	r = −0.30, *p* = 0.302	r = −0.08, *p* = 0.782
Score	r = 0.02, *p* = 0.937	r = −0.44, *p* = 0.117	r = −0.14, *p* = 0.628	r = 0.17, *p* = 0.562
WCST error rate	r = −0.14, *p* = 0.574	r = −0.11 *p* = 0.703	r = 0.02, *p* = 0.935	r = −0.11, *p* = 0.714
RT (s)	r = −0.21, *p* = 0.397	r = −0.25, *p* = 0.391	r = −0.39, *p* = 0.164	r = 0.25, *p* = 0.391

## Discussion

The hypothesis that (I) low-reinvesters have less disrupted sleep than high-reinvesters must be rejected. (II) Athletes with less disrupted sleep have greater cognitive performance capacity in terms of RT when recalling memory, however not in performance outcomes; therefore the hypothesis can only be partly accepted, and must partially be rejected. (III) Perceived sleep quality and physical health, as well as (IV) perceived stress scores did not correlate with athletes' sleep measures, only wellbeing (mental health) correlated with total sleep-time; the hypothesises in this regard must therefore partly be rejected, and can only be partly accepted. However, athletes with longer durations of SD (awake) time during night sleep were slower in recalling memory function, yet performance outcome was not affected in the Backwards Corsi test. Participants with greater reported physical health whatsoever had more error-rates in terms of cognitive flexibility, indicating that athletes who did not feel good physically performed better in the WCST. Individuals with greater perceived physical health also had less tendency to ruminate and reinvest in their decisions, as elicited by lower DSRS scores. Neither of these measures correlated with overall scores and mean RT of the STROOP colour-word test.

A major limitation of this study was the variety of devices and techniques used to record sleep data. Even though more than half of the participants (*N* = 11) used the same type of tracker, functionality and sensitivity may alter among different methods (45). The reliability of consumer trackers for healthy sleepers is reasonably good in comparison to the golden standard PSG (45, 46). Also, no standardised test setting could be ensured, when performing the test battery on PsyToolkit. However, a recent study of Barcellos et al. (55) validated the reliable use of remote cognitive assessments in individuals with multiple sclerosis, and claims similar results in comparison to clinical settings. According to the Individual Zone of Optimal Function (IZOF) theory, performance relies among other things, on the situational emotional experiences (27); indicating if individuals do not feel great in a certain (i.e., clinical) setting, they might not perform as great as they would in a more engaging setting, in accordance to their individual needs. Under this aspect, the remote testings might be considered an advantage, that is overcoming the limitations of clinical settings. Furthermore, time of sleep onset each day, and time of waking each day, were either not properly provided by all individuals, or participants had additional professional obligations with shift-work, that challenged comparability of the data. However, all participants had additional professional obligations to their sports. Athletes participating the first league were all females, and contractually not obliged to the same extend as professional males; and therefore comparable to males and females in semi-professional leagues. Participants using Apple Watch had delayed sleep onset and less time recorded in bed than sleeping, when for example falling asleep on the couch and then going to bed later on. Time of sleep onset and wakings were therefore excluded from the analysis. At-home validation (i.e., with mobile PSG) of consumer trackers is recommended, rather than clinical validation were athletes do not get to fall asleep on the couch watching Netflix. Also, years of professional experience had not been determined in participants. “Anxiety is related inversely with the amount of time spent practising, with experience[,] and with the number of competitions in which the athletes participated …” (56); therefore experienced players can better cope with competitive demands, and might not be significantly affected by differences in sleep, and stress induced performance decline. The number of participants was based on the Hansen et al. (48) study, that found differences in performance and sympathetic response of sleep deprived individuals. Due to the explorative nature of the study, no sample size computation was done. This is a limitation of the study which does not affect the significant effects and relations. It is likely that additional effects will be found if the sample size is increased, consequently we suggest follow-up studies to further analyse this field of research. The number of recorded nights however was chosen with respect to Svensson's et al. (46) validation process, comparing Fitbit devices against portable single-channel EEG systems.

### SD time, selective attention, and memory reaction time

This study can not confirm any relationship between both STROOP mean RT and overall scores, with sleep quality in football players. Both anticipation skills and memory are critical pillars of adequate decision making in sports, and require great amount of attention (3). The STROOP colour-word test assesses selective attention, and is therefore a reliable predictor for anticipatory response and memory function in athletes. However, previous research has demonstrated generally greater visual-motor performance in high-level athletes compared to non-athletes (57), why potential minor differences in sleep quality, might not necessarily correlate with performance outcomes; as within the scope of total sleep deprivation (58), or partial sleep deprivation (59).

Memory function in athletes was not affected by sleep quality in this research either, yet athletes whose sleep was disturbed at night for longer periods of time had longer RT processing and recalling memory in the Backwards Corsi test. However, the prefrontal cortex review, on single neurone level, of Lara and Wallis (60), came to the conclusion that delayed period activity in the prefrontal cortex, might be a top-down signalling that influences the posterior sensory areas in the brain, that are suggested by the authors to be actually responsible for running working memory functions. Still, the role of trait and state related anxiety, and attention control triggering a bottom-up control remains not covered in this context. Athletes also outperformed non-athletes in a study of Barhorst-Cates (61), performing an analogue Corsi task and a new full-bodied version of the Corsi task, called the “Twister Task.” Barhorst-Cates suggests that athletes possess superior spatial working memory capacity, and therefore be potentially more resilient to minor differences in sleep quality, in regard of their memory performance. Delayed RT however might be a goal-oriented coping mechanism, rather than an bottom-up response to stress. Several muscle twitches per hour occur during sleep in healthy individuals, and act against muscle atonia. Muscle twitches derive from brain areas that normally do not fire during movement, namely the hippocampus, cellebral cortex, and red nucleus (62). Interestingly, memory consolidation processes (63), as well as overnight improvement of spatial navigation (64); are strongly associated to initially dependent on hippocampal activity, and then become hippocampus-independent over night. Increasing motor activity predicts the end of sleep towards wakefulness, and may trigger and promote arousal from REM sleep (65). The ability to be undisturbed by these triggers relies on glutamatic sub-laterodorsal activity; that if disturbed, produces REM sleep without muscle atonia and reduced total REM time (42). The Desseilles paper further states in their review that, awaking and partial arousals are often accompanied by random sleep myoclonus (muscle contractions), and known as Periodic Limb Movements in Sleep (PLMS), or Restless Legs Syndrome (RLS), when becoming more frequent and regular. According to the authors, both PLMS and RLS derive for brain activity in the cerebellum and thalamus, and are accompanied by activity in the red nuclei and brainstem; and often occur in individuals with anxiety and depression. Excessive motor activity during sleep however is often also linked to consumption of stimulants, both physical (i.e., hard exercise before sleep) and emotional stress, sleep deprivation, or neurodegenerative diseases (66). Mental health scores whatsoever did not correlate with awake times or RT in any of the cognitive assessments in this research. Mental wellbeing only seems be related to the total amount of sleep. In order for PLMS and RLS to be diagnosed, individuals require some sort of sleep complaint, and the presence of motor activity during PSG that can be excluded to derive from other causes. Sleep myoclonus however might not be the only reason why athletes wake up at night. Dehydration, bladder emptying, odour, noise, or any other sensory response, i.e., to pets and bed-partners, might be triggering sleep interruptions (64). More comprehensive assessments of individuals, and qualitative interviews can promote identifying underlying causality of frequently occurring and enduring sleep interruptions.

As previously mentioned, REM sleep was not recorded in all participants in this study, and therefore limits the comparability of RT in memory recalling, as SD times can not be compared to REM times. Also, there were only 14 available data points for the comparison of SD time and Corsi RT, that were captured by five different devices. Nevertheless, REM sleep plays a critical role in the formation and consolidation of certain types of memory (42), by spiking calcium levels within dendrites for strengthening new spines, coordinated restoration and elimination of nerve cells, receptors, and synapsis, promoting cortical plasticity (67). According to the Peever review, REM SD impairs both the spatial and emotional memory. Meanwhile, the general relationship between REM sleep and memory function is being challenged by the animal kingdom. The amount of REM sleep across species depends on both brain- and body-mass. The Peever review further states that, elephants spend very little time in REM, and yet have superior memory skills in comparison with other animals. Time spent in REM sleep is also determined by ecological and environmental factors (42), as greater levels of stress and alertness result in less REM time. Peever concludes that REM time however increases towards the end of sleep, and therefore raises suspicion that longer periods of total sleep might be able to compensate for poor sleep quality. Nevertheless, there are 3 types of fatigue: transient (1–2 days), cumulative (several days), and circadian (68). Even though athletes roughly slept the same amount in this study, this study did not assessed whether participants perceived that the amount slept is covering their individual physiological and psychological demands, and whether they suffers some sort of fatigue.

### Perceived health, cognitive flexibility and reinvestment

Sleep quality however could not be correlated to reinvestment, in this study, and might potentially not be the root cause for greater sympathetic baseline activity in high-reinvesters. Nevertheless, participants with greater physical health scores in the SF-36 assessment had more error-rate, in terms of cognitive flexibility, in the WCST; yet, self-reported physical health also negatively correlated with reinvestment scores in this study. These findings are indicating that athletes who did not feel good physically, and therefor had lower physical health scores, performed better in the WCST. Athletes who rated their physical health lower, rated their tendency to ruminate and reinvest in their decisions higher instead. Perceived physical health might potentially play a greater role on reinvestment, than previously estimated. Whatsoever, there was no correlation between reinvestment scores and error-rates in the WCST. This paradoxum might be in response to both the STROOP and reinvestment effect. Greater sympathetic activity, both at rest and under pressure, has recently been linked to grater tendency to ruminate and reinvest in decisions (11); and performance differences in high-reinvesters have previously been observed under pressure (11, 23). The study of Gomez et al. (16) confirmed increased cortisol levels and sympathetic activity in response to the STROOP colour-word test; which in this research was conducted prior to the WCST. Therefore, the WCST can be considered a pressured task, and might have triggered reinvestment behaviour in high decision-reinvesters. One of the key attributes of high-reinvesters, is that these individuals do not stick to learned behaviour under pressure (11, 22, 23, 43); which in the WCST has been an advantage to perform better, in this research. A potential explanations could be that, individuals with less self-reported physical health scores, that significantly also had greater tendency to ruminate and reinvest, had shown greater cognitive flexibility when attempting to find new strategies in the WCST. However, previous studies have shown conflicting evidence in regard of cognitive performance in high-reinvesters (11, 12). Non-athletes might potentially have a lower stress thresholds and be affected by Hardy and Fazey's Chaos theory much faster than athletes. Athlete reinvesters potentially benefit in response to reinvestment accompanied sympathetic activity, from exercise induced vagal activity (69), and are therefore not affected by physiological differences to the same extend as non-athlete high-reinvesters.

Regardless of all theses hypothesises and theories, athletes that did rate their reinvestment scores higher in the DSRS, for some reason perceive that something is not optimal with their health, in this research. Questions in regard of physical health covered (I) physical function, (II) pain, (III) general health, and (IV) the role of physical health limiting other activities. Therefore, athletes perception might be in regard of underlying physiological issues slumbering under the surface, that are not fully developed until triggered upon a certain threshold, in order to call sick on the pitch (70). The cohort study of Timpka et al. (70) during the Beijing 2015 World Championship of the International Association of Athletics Federations, came to the conclusion that athletes with increased pre-participation anxiety levels, in regard of their injury and illness symptoms, were at greater risk to be injured during competition. The researches analysed 957 Pre-participation Health Questionnaires (PHQ), with actual injury and health outcomes after the championship. The authors further state that “preparticipation [sic] symptoms causing anxiety are interesting predictors for in-championship health problems” and that therefore “… endurance athletes require particular clinical attention.” The Austrian physician, neurophysiologist, and pioneer of the psychoanalysis Sigmund Freud states in his work (71) in this regard that “the issues are trapped in the tissues”; referring to his observation that every psychological condition is likely to be linked to an underlying physiological cause. Therefore, regularly including perceived estimations of individuals, when assessing athletes health status, might not only prevent injuries and illnesses, but also reduce tendency to rumination and reinvest in the execution of automated skills during competition, especially when under pressure. Yet, for the purpose of the WCST athletes with lower physical health scores, probably benefited from greater reinvestment tendencies in this research; as literature in regard of physiological stress and mental alertness (68) is conflicting the potential theory that individuals with lower physical health scores might have been mentally more alert.

In order to answer the question of whether athlete high-reinvesters have greater sympathetic response than athlete low-reinvesters, and whether sympathetic response is different from non-athlete high-reinvesters; further studies might consider the use of AIC scans to asses brain activity in regard of bodily sensation (7); but also inflammatory markers such as interleukins (IL) 1, 6, 17 that act on the HPA axis and promote cortisol secretion (17), as well as Angiotensin II (72); both at rest and under pressure. The cross-sectional research of Bascour-Sandoval et al. (73) examined sleep behaviour of 71 male and female amateur athletes, conducting the Pittsburgh Sleep Quality Index (PSQI) questionnaire, with respect to self-reported pain levels on a Numerical Rating Scale (NRS). The study claims that individuals with increased pain levels both at rest and during sports, also had decreased sleep quality (lower scores on the PSQI). Even though SD time did not correlate with perceived health in this study, underlying physiological conditions, such as low threshold pain levels, that athletes cope well with, might be unconsciously affecting athletes' sleep and overall physical wellbeing. Interestingly, SD time did not correlate with ASBQ scores either. Athletes who woke up a lot during bed-time did not have greater ASBQ scores. In fact the ASBQ does not cover questions in regard of sleep interruptions, and might therefore require revision. An other aspect that has previously shown to enhance both physiological and psychological performance indicators in a pilot study, is mindfulness training (74). The effect of 7 weeks structured mindfulness training was tested in a repeated measure study against a control group. Sparks et al. (75) later added to that finding and came to the conclusion that especially high reinvesters benefit from mindful refocus the most. The researchers compared 270 rowers in a cross-sectional study, and examined post-competition scores of perceived performance, anxiety, mindfulness, and reinvestment in comparison to actual performance. Basically, Sigmund Freud's psychoanalysis, which is a clinical tool to address psychopathology, by adapting inner dialog, reframing, and language could be considered the roots of mindfulness. Experimental studies may want to take Freud's and Sparks' finding in regard of physiological adaptations to mindfulness into account, when examining high-reinvesters in the future.

## Conclusion

In summary, the research found evidence that, athletes with longer SD (awake time) during night sleep were slower in processing and recalling memory, in the Backwards Corsi test. Delayed reaction potentially indicate a successful coping mechanism, as athletes' performance was not affected. Athletes superior spatial and reactive skills however might not be affected by minor differences in sleep quality, to the same extend as individuals in studies with total or partial sleep deprivation. Yet, SD alone is not telling the whole story, and assessment of REM sleep–awake time ratios, as well as qualitative assessments are recommended for further researches. Also, at-home validation of consumer trackers is required, as tracker came up with conflicting recordings in individuals who felt asleep on the couch before actually going to bed. Sleep quality whatsoever, could not be linked to greater tendency to reinvest, but wellbeing (mental haealth) was increased in individuals with greater amount of total sleep time. Athletes who did not feel good physically (lower physical health scores in the SF-36 questionnaire) performed better in the Wisconsin Card Sorting Test, while pain negatively influenced memory function. Individuals with lower physical health scores also had greater tendency to ruminate and reinvest in their decisions, as elicited by higher Decision Specific Reinvestment Scale scores. High-reinvesters therefore might require more clinical attention, to identify underlying physiological issues. Reinvestment *per se* however could not be linked to performance differences. Athlete reinvesters potentially cope better with competitive concerns due to exercise induced vagal activity, than non-athlete reinvesters. Selective attention in terms of overall scores and mean RT of the STROOP colour-word test could not be linked to either measure, but likely to trigger reinvestment behaviour.

## Data availability statement

The original contributions presented in the study are included in the article/Supplementary material, further inquiries can be directed to the corresponding authors.

## Ethics statement

The studies involving human participants were reviewed and approved by Science and Engineering Research Ethics and Governance Committee of the Manchester Metropolitan University, under the reference number 25719. The patients/participants provided their written informed consent to participate in this study.

## Author contributions

JP: designed the study, collected data, analysed data, and wrote paper. JS and KR: supervisor to first author. WH: responsible statistician. All authors contributed to the article and approved the submitted version.
